# Regional Nerve Blocks for Trauma Pain in the Emergency Department: A Systematic Review of Efficacy and Safety

**DOI:** 10.7759/cureus.82073

**Published:** 2025-04-11

**Authors:** Jalal Abu Halimah, Ali A Zalah, Arwa H Alammari, Shorog B Basowed, Abdullkarim T Mobarki, Ethar A Khawaji, Revan A Arishi, Rawan S Almohammed, Alzahra A Almubarak, Bayan A Buhulaigah

**Affiliations:** 1 General Surgery, Jazan University, Jazan, SAU; 2 Medicine, Jazan University, Jazan, SAU; 3 Diagnostic Imaging, Security Forces Hospital, Dammam, SAU; 4 General Practice, Prince Mohammed Bin Nasser Hospital, Jazan, SAU; 5 Medicine, University of Tabuk, Tabuk, SAU; 6 Medicine and Medical Sciences, Arabian Gulf University, Manama, BHR

**Keywords:** acute pain management, emergency department, musculoskeletal injuries, opioid reduction, patient satisfaction, procedural sedation, randomized controlled trials, regional anesthesia, trauma, ultrasound-guided nerve block

## Abstract

Effective acute pain management in the emergency department is essential, especially for patients with fractures, dislocations, and other musculoskeletal injuries. Conventional approaches like systemic opioids and procedural sedation, while commonly used, can lead to side effects such as respiratory issues, nausea, extended emergency department stays, and potential opioid dependence. In recent years, ultrasound-guided nerve blocks have gained attention as a safer and more targeted method of pain control that can reduce reliance on opioids. This review explored how ultrasound-guided nerve blocks compare to traditional pain management strategies in terms of effectiveness, safety, and clinical outcomes. A detailed search was conducted across several major medical databases, including PubMed, Scopus, Web of Science, the Cochrane Central Register of Controlled Trials, and the Virtual Health Library. Search terms focused on ultrasound, anesthesia, nerve blocks, and emergency care. The review included only randomized controlled trials published in English that involved adult patients receiving ultrasound-guided nerve blocks for acute pain in emergency settings. The main outcomes assessed were pain relief (measured using validated scales), opioid use, time spent in the emergency department, patient satisfaction, and adverse events. Out of 3,299 studies initially identified, 2,430 remained after removing duplicates. After reviewing 60 full-text articles, nine met the inclusion criteria for analysis. The results showed that ultrasound-guided nerve blocks provided more rapid and sustained pain relief than standard treatments, with a noticeable reduction in opioid use. They were also linked to shorter emergency department stays, higher levels of patient satisfaction, and a low rate of complications when carried out by trained providers. These findings suggest that ultrasound-guided nerve blocks can be a highly effective first-line option for managing acute pain in trauma patients. Still, further studies are recommended to optimize their use and encourage broader implementation in emergency care settings.

## Introduction and background

Effective pain management is a cornerstone of trauma care in the emergency department. Traditionally, systemic analgesics, including opioids, have been the mainstay for alleviating acute pain. However, concerns regarding opioid-related adverse effects, such as respiratory depression and the potential for dependence, have prompted the exploration of alternative analgesic modalities [1]. Ultrasound-guided regional nerve blocks have emerged as a promising technique, offering targeted pain relief while potentially mitigating the risks associated with systemic analgesia [2].

The integration of ultrasound-guided regional nerve blocks into emergency department practice represents a significant advancement in acute pain management [3]. Ultrasound guidance enhances the precision and safety of nerve block procedures by providing real-time visualization of neural structures. This technique allows for accurate needle placement and optimal local anesthetic distribution, thereby improving block success rates and reducing the likelihood of complications [3].

Clinical studies have demonstrated the efficacy of ultrasound-guided regional nerve blocks in reducing pain scores among trauma patients [1]. For instance, a study observed a significant decrease in pain scores from 7.4 to 2.8 following the administration of ultrasound-guided regional nerve blocks, highlighting their potential in managing acute pain effectively [4]. Moreover, the implementation of ultrasound-guided regional nerve blocks has been associated with a reduction in opioid consumption, addressing concerns related to opioid overuse and its associated complications [4].

The safety profile of ultrasound-guided regional nerve blocks is also noteworthy. A recent study reported that 70.8% of ultrasound-guided regional nerve blocks were successful in achieving pain reduction of 51% to 100%, underscoring both their efficacy and safety when performed by trained practitioners [5]. Additionally, the American College of Emergency Physicians has recognized ultrasound-guided regional nerve blocks as a core skill for emergency physicians, advocating for their use as part of a multimodal pain management approach in the emergency department [6].

Despite the growing body of evidence supporting ultrasound-guided regional nerve blocks, their adoption in emergency settings varies, and concerns about potential complications persist. Therefore, a systematic review of the efficacy and safety of ultrasound-guided regional nerve blocks for trauma pain in the emergency department is warranted. Such a review would provide a comprehensive analysis of current evidence, guiding clinical practice and informing policy development to optimize pain management strategies in trauma care.

## Review

Methods

Literature Search Strategy

This systematic review was registered with PROSPERO, the International Prospective Register of Systematic Reviews. We adhered to the Preferred Reporting Items for Systematic Reviews and Meta-Analyses (PRISMA) guidelines during the conduct and reporting of this review. Our comprehensive search strategy included four major online databases: PubMed, Web of Science, Scopus, and the Cochrane Central Register of Controlled Trials (CENTRAL), as well as the Virtual Health Library (VHL), covering publications from inception until March 5, 2025.

We employed a structured Boolean search strategy to ensure the identification of relevant studies, using the following search terms: ("Ultrasound" OR "Ultrasound-guided") AND ("Anesthesia" OR "nerve block") AND ("Emergency" OR "emergency department"). Each database's search strategy was tailored to maximize sensitivity and specificity in identifying randomized controlled trials evaluating ultrasound-guided nerve blocks for emergency pain management. Filters were applied to include only studies published in English, involving human participants, and clinical trials to maintain a high level of methodological rigor.

To enhance the completeness of our review and reduce publication bias, we conducted a manual reference screening of the included articles, ensuring that any additional relevant studies not captured by the initial database search were identified and included. This rigorous approach ensures that the systematic review comprehensively evaluates the current evidence on ultrasound-guided nerve blocks for acute pain management in emergency settings.

Eligibility Criteria

The selection criteria were based on the population, intervention, comparison, outcome, and study design framework to ensure a rigorous and structured inclusion process for evaluating ultrasound-guided nerve blocks in emergency pain management. We included only randomized controlled trials published in English that involved adults aged 18 years and older presenting to emergency departments with acute pain conditions from trauma or migraine. Studies are needed to assess the effectiveness of ultrasound-guided nerve blocks, including femoral nerve blocks, interscalene brachial plexus blocks, sciatic-femoral nerve blocks, pericapsular nerve group blocks, and greater occipital nerve blocks. Additionally, studies had to compare these nerve blocks with standard pain management strategies, such as intravenous opioids (morphine, fentanyl, tramadol), procedural sedation (propofol, etomidate, fentanyl), general anesthesia, or non-opioid analgesics (dexketoprofen, paracetamol, tramadol). Outcome measures needed to include pain reduction (measured by numerical rating scale or visual analog scale), opioid consumption, emergency room length of stay, patient satisfaction, and adverse events.

We excluded studies that were not randomized controlled trials, such as observational studies, case series, and retrospective reviews. Studies involving pediatric patients (under 18 years), those without a comparator group, and those that did not use ultrasound guidance for nerve block administration were also excluded. Additionally, research focused on chronic pain management, elective surgery, post-operative analgesia, or non-acute settings was excluded. Non-English studies, conference abstracts without full-text availability, and trials investigating epidural or spinal anesthesia instead of peripheral nerve blocks were omitted from the review.

Study Selection

Two reviewers independently screened the titles and abstracts of the retrieved articles using predetermined eligibility criteria. Any disagreements or discrepancies were resolved by a third reviewer until consensus was reached. The full text of the included articles was further analyzed, and the following data were extracted: sample size, type of telemedicine technology used, age of participants, outcome measures, and main results. A third reviewer resolved any potential conflicts.

Quality Appraisal

The methodological quality of the included studies was independently assessed by two reviewers using the modified Downs and Black scale for clinical trials [7]. The scale consists of 27 questions rating four categories: reporting, external validity, internal validity, and power. Studies were considered to be of excellent quality when the final score ranged from 26 to 28, good quality if the score ranged from 20 to 25, fair quality if the score ranged from 19 to 15, and poor if the score was 14 or less. Any disagreements or discrepancies were resolved by discussion until a consensus was reached.

Search Strategy

The search strategy employed for this systematic review involved five major databases: PubMed, Scopus, Web of Science, CENTRAL, and the VHL Regional Portal. The search terms used were ("Ultrasound" OR "US") AND ("Anesthesia" OR "nerve block") AND ("Emergency" OR "ED"), ensuring a comprehensive retrieval of relevant studies. This systematic search yielded a total of 3,299 records across all databases: PubMed (437), VHL Regional Portal (140), CENTRAL (758), Web of Science (1,023), and Scopus (941). Following duplicate removal, 2,430 studies remained for screening. Titles and abstracts were independently reviewed, excluding 2,370 studies that did not meet the eligibility criteria. The remaining 60 full-text articles underwent detailed evaluation, with 51 studies excluded due to incorrect population, intervention, comparator, or study design. The final selection included nine studies [8-16] that met all inclusion criteria, which were subsequently included in the qualitative analysis.

**Figure 1:**
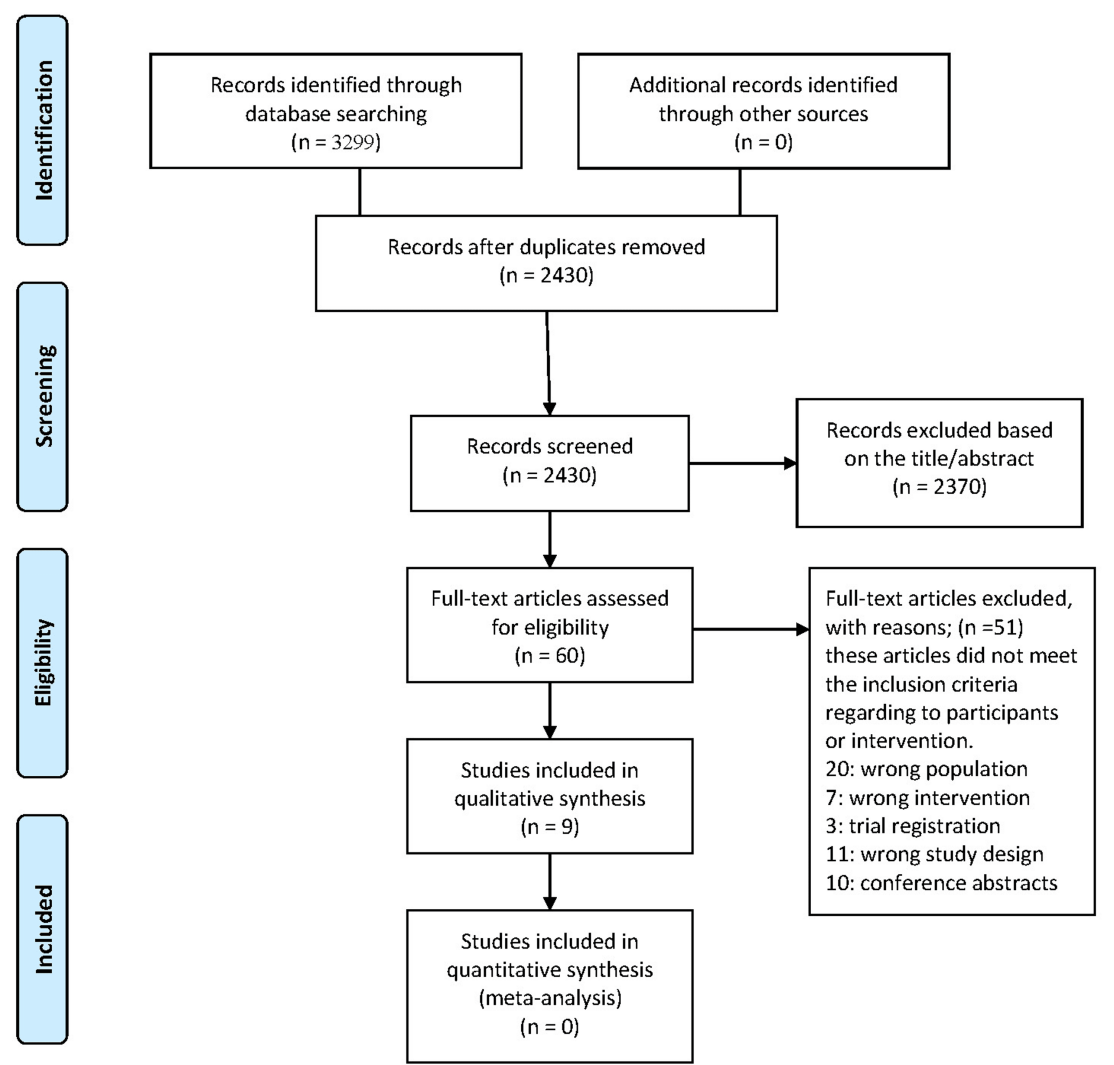
Flow diagram of the study selection process according to PRISMA guidelines PRISMA: Preferred Reporting Items for Systematic Reviews and Meta-Analyses

The excluded studies comprised those that assessed the wrong population, inappropriate interventions, or study designs that did not align with the review criteria. Only randomized controlled trials were retained, investigating ultrasound-guided nerve blocks for acute pain management in emergency settings. The final set of nine studies included in this systematic review was subjected to qualitative synthesis, ensuring high methodological rigor and reliability. The PRISMA flowchart in Figure 1 visually represents the study selection process, from database searching to final inclusion.

Results

Study Characteristics

All the included studies were randomized controlled trials conducted in various emergency department settings across multiple countries, including the United States [8,9,13], the United Kingdom [14], France [11], South Korea [10], Turkey [12,16], and Iran [15]. The studies included different hospital levels, from level I trauma centers [9] to university medical centers [10,15] and district general hospitals [14]. The sample sizes varied between 30 and 60 participants, with an approximately equal distribution of male and female patients in most studies, although some trials exhibited a gender imbalance. For instance, Beaudoin et al. [8] and Gerlier et al. [11] had higher female representation (73-86% in some groups) due to the higher incidence of hip fractures in elderly women, whereas Kang et al. [10] and Mutty et al. [13] had predominantly male participants (65-85%) due to the nature of lower limb fractures often resulting from high-impact trauma (Table 1).

**Table 1:** Study characteristics and outcomes FNB: femoral nerve block, SC: sciatic nerve block, US: ultrasound, PSA: procedural sedation and analgesia, ISBPB: interscalene brachial plexus block, IVT: intravenous therapy, GONB: greater occipital nerve block, PENG: pericapsular nerve group block, FN: femoral neck, IT: intertrochanteric fracture, FT: femoral shaft fracture, SG: subglenoid dislocation, MME: morphine milligram equivalent, VAS: visual analog scale, NRS: numeric rating scale, ERLOS: emergency room length of stay, LOS: length of stay, SPID: sum of pain intensity differences

Study ID	Country	Design	Setting	Sample size and demographics	Type of trauma	Type of nerve block	Anesthetic agent	Comparator	Outcome measures	Study results and key findings
Beaudoin et al., 2013 [8]	USA	RCT	ED, Rhode Island Hospital	N = 36 (FNB: 18, SC: 18), age: 82 (64–98), female (%): FNB: 61%, SC: 81%	Hip fracture (FN, IT)	US-guided 3-in-1 FNB	Bupivacaine 0.5% (25 mL)	IV opioids (morphine)	SPID over 4 hours, rescue analgesia, adverse events	NRS at 4 hours: FNB: 4.0, SC: 8.0 (p < 0.001), SPID: FNB: 11.0, SC: 4.0 (p = 0.001), rescue morphine: FNB: 0.0, SC: 5.0 (p = 0.028), no significant adverse events
Blaivas et al., 2011 [9]	USA	RCT	Level I trauma ED	N = 42 (PS: 21, ISB: 21), age: PS: 35.9, ISB: 39, female (%): PS: 29%, ISB: 24%	Shoulder dislocation	US-guided ISB	Lidocaine + epinephrine (20–30 mL)	Procedural sedation (etomidate)	LOS, provider time, pain (VAS), satisfaction, complications	LOS: PS: 177.3 min, ISB: 100.3 min (p < 0.0001), provider time: PS: 47.1 min, ISB: 5 min (p < 0.0001), no difference in pain, satisfaction, or complications
Kang et al., 2017 [10]	South Korea	RCT	Chungnam Nat’l Univ. Med Center	N = 40 (USNB: 20, GA: 20), age: USNB: 55.6, GA: 54.2, male (%): USNB: 85%, GA: 75%	Lower leg fractures (AO 42–44)	US-guided femoral + sciatic	Lidocaine 1% (30 mL) + ropivacaine 0.75% (20 mL)	General anesthesia	Lead time, satisfaction, anesthesia preference, complications	Lead time: USNB: 4.3 hours, GA: 9.4 hours (p < 0.001), satisfaction: USNB: 9.0, GA: 4.9 (p < 0.001), all USNB patients preferred NB
Gerlier et al., 2024 [11]	France	RCT	ED, Paris Saint-Joseph Hospital	N = 30 (FNB: 15, SC: 15), age: FNB: 87.9, SC: 85.3, female (%): FNB: 73.3%, SC: 86.7%	Hip fracture (FN, FT)	US-guided FNB	Ropivacaine 7.5 mg/mL (20 mL)	IV morphine titration	Opioid use, time to relief, adverse events, procedure ease	48h opioid use: FNB: 6 MME, SC: 15 MME (p < 0.001), 40% fewer opioid-related AEs in FNB, no difference in time to relief
Güllüpınar et al., 2022 [12]	Turkey	Prospective RCT	ED, İzmir Bozyaka Training and Research Hospital	N = 39 (PENG: 18, control: 21), age: 75.3 ± SD, male (%): 66.7%	Hip fracture (FH, IT, ST, FN)	PENG block	Bupivacaine 0.25% (20 mL)	IV paracetamol/tramadol	Pain (NRS), systemic analgesia, adverse events, satisfaction	Pain at 30 min: PENG: 1.78, control: 3.38 (p = 0.01), pain at 6 hours: PENG: 0.00, control: 2.86 (p < 0.001), PENG reduced systemic analgesia need
Mutty et al., 2007 [13]	USA	RCT	ED, Erie County Medical Center	N = 54 (FNB: 31, SC: 23), age: FNB: 42, SC: 45, male (%): FNB: 65%, SC: 70%	Femoral fractures (diaphyseal, distal)	Femoral nerve block	Bupivacaine 0.5% (20 mL)	IV narcotics + traction splint	Pain scores (VAS), complications	Pain at 5 minutes: FNB: 4.9, SC: 8.2 (p < 0.001), pain at 90 minutes: FNB: 3.5, SC: 7.0 (p < 0.001), no major complications
Fletcher et al., 2003 [14]	UK	RCT	District General Hospital ED	N = 50 (FNB: 26, IVM: 24), age: FNB: 76, IVM: 80, female (%): FNB: 71%, IVM: 69%	Hip fracture (FN)	3-in-1 FNB	Bupivacaine 0.5% (20 mL)	IV morphine	Time to lowest pain, opioid consumption, pain scores, complications, mortality	Faster pain relief: FNB: 2.88 hours, IVM: 5.81 hours, lower pain scores in FNB group, 6-month mortality equal
Raeyat Doost et al., 2017 [15]	Iran	RCT	ED, Bahonar Hospital	N = 60 (ISBPB: 30, PSA: 30), age: 28.7 ± 7.7, male (%): 96.7%, dislocation type: SC: 60%, SG: 40%	Anterior shoulder dislocation	US-guided ISBPB	Lidocaine 1% (15–25 mL) + epinephrine (1:100,000)	Procedural sedation (propofol + fentanyl)	ERLOS, pain (NRS), reduction attempts, satisfaction, complications	ERLOS: ISBPB: 80.2 minutes, PSA: 108.6 minutes (p = 0.005), pain reduction: ISBPB: 3.43, PSA: 0.38 (p < 0.001), PSA had higher satisfaction
Korucu et al., 2018 [16]	Turkey	RCT	ED, Kecioren Training and Research Hospital	N = 60 (GONB: 20, IVT: 20, placebo: 20), age (median): GONB: 40, IVT: 35, placebo: 40, male (%): GONB: 10%, IVT: 25%, placebo: 10%	Acute migraine	Greater occipital NB	Bupivacaine 0.5% (1 mL) + saline (1 mL)	IV dexketoprofen + metoclopramide, placebo	Pain (PSS), pain reduction, adverse events	Pain at 30 minutes: GONB: 3, IVT: 1, placebo: 4.5 (p = 0.01), no severe adverse events

Most studies focused on acute pain management following trauma, particularly hip fractures, shoulder dislocations, and lower extremity fractures. Hip fractures [8,11,12,14] were the most frequently studied injury type, given the high prevalence of hip fractures in older adults and the associated pain that requires urgent pain control. These studies specifically examined femoral neck and intertrochanteric fractures, common in elderly patients following falls. Shoulder dislocations [9,15] were also a key area of interest, as they require immediate reduction and often involve severe acute pain, making nerve blocks an attractive alternative to procedural sedation. Additionally, lower extremity fractures [10,13] were evaluated, particularly diaphyseal and distal femoral fractures, which can be extremely painful and require prolonged immobilization. Korucu et al. [16] was the only study investigating a non-traumatic pain condition (acute migraines) using a greater occipital nerve blockade, highlighting the potential expansion of nerve block applications beyond orthopedic injuries.

The type of nerve block used varied significantly across studies, depending on the type of trauma being treated. Femoral nerve blocks were the most commonly used blocks in studies evaluating hip and femoral fractures [8,11,13,14]. These blocks target the femoral nerve, providing effective analgesia to the anterior thigh and knee and reducing the need for systemic opioids. Interscalene blocks were utilized [9,15] to anesthetize the brachial plexus for shoulder dislocations, effectively numbing the shoulder region. Pericapsular nerve group blocks were explored by Güllüpınar et al. [12] as a potentially superior alternative to femoral nerve block in hip fractures, as pericapsular nerve group blocks specifically target the articular branches innervating the hip joint, providing more targeted pain relief with potentially fewer motor impairments. Sciatic and femoral nerve blocks were combined [10] for lower extremity fractures, ensuring widespread analgesia for both the anterior and posterior compartments of the leg. In migraine treatment [16], greater occipital nerve blocks were used to interrupt pain pathways in patients suffering from acute migraine attacks.

The anesthetic agents used in these studies primarily included bupivacaine, which was used in various concentrations (0.25-0.5%) and volumes ranging from 20-30 mL, depending on the procedure. Bupivacaine was the most preferred local anesthetic due to its long duration of action, making it ideal for post-procedural pain relief in trauma patients. Some studies used ropivacaine, a safer alternative with reduced cardiac toxicity, in cases such as Gerlier et al. [11] and Kang et al. [10], where prolonged analgesia was needed without excessive motor block. Lidocaine, often combined with epinephrine, was used in shorter-duration procedures, such as shoulder dislocations [9,15], as the epinephrine component prolongs the local anesthetic’s duration while reducing systemic absorption. Korucu et al. [16] employed a small dose of bupivacaine combined with saline in greater occipital nerve blocks for migraine treatment, demonstrating that even a minimal amount of anesthetic could provide significant pain relief in non-traumatic conditions.

The comparator interventions varied widely across studies and reflected each condition's standard pain management practices. IV opioids such as morphine were the most commonly used comparators in hip fracture and femoral fracture studies [8,11,13,14], as they represent the current standard of care in acute pain relief. Procedural sedation was used as a comparator in studies examining shoulder dislocations [9,15], utilizing agents such as etomidate, propofol, and fentanyl to facilitate joint reduction. Kang et al. [10] compared nerve blocks to general anesthesia, assessing their effectiveness in reducing preoperative pain and shortening the time to surgery. Non-opioid analgesics, such as dexketoprofen and tramadol, were used as comparators in Korucu et al. [16] and Güllüpınar et al. [12], highlighting the potential for nerve blocks to replace systemic analgesics in non-traumatic pain conditions.

Quality Assessment

The quality assessment of the included studies demonstrates that all were randomized controlled trials with moderate to high methodological rigor, scoring between 21 and 24 points. Reporting quality was strong across all studies, with most scoring 8 or 9 out of 10, indicating well-documented methodologies, interventions, and outcome measures. Gerlier et al., Kang et al., and Güllüpınar et al. scored the highest in reporting (9 points) [10-12], suggesting comprehensive study protocols, while Blaivas et al., Fletcher et al., Mutty et al., and Raeyat Doost et al. [9,13-15] scored slightly lower (8 points), likely due to minor gaps in randomization procedures or intervention fidelity descriptions. These findings confirm adequate transparency in methodology across all trials, ensuring reproducibility and clarity (Table 2).

**Table 2:** Methodological quality assessment of included studies This table summarizes the methodological quality of included studies, evaluated using the Downs and Black checklist [7] for assessing the quality of randomized and non-randomized health care intervention studies. The checklist consists of five domains: reporting (10 items), external validity (3 items), bias (7 items), confounding (6 items), and power (1 item). Each study was assessed for these domains, and the scores are reflected in the table. The total score is the sum of the scores across all domains, with a higher score indicating better methodological quality. Scores range from 0 to a maximum of 27.

Study ID	Reporting (10 items)	External validity (3 items)	Bias (7 items)	Confounding (6 items)	Power (1 item)	Total score
Beaudoin et al., 2013 [8]	9	2	5	4	1	21
Blaivas et al., 2011 [9]	8	2	6	4	1	21
Kang et al., 2017 [10]	9	2	6	5	1	23
Gerlier et al., 2024 [11]	9	3	6	5	1	24
Güllüpınar et al., 2022 [12]	9	2	6	5	1	23
Mutty et al., 2007 [13]	8	2	6	5	1	22
Fletcher et al., 2003 [14]	8	2	6	4	1	21
Raeyat Doost et al., 2017 [15]	8	3	6	4	1	22
Korucu et al., 2018 [16]	9	3	5	4	1	22

External validity scores varied between 2 and 3 points, indicating differences in how well the findings could be generalized to broader populations. Studies such as Gerlier et al., Korucu et al., and Raeyat Doost et al. [11,15,16] received the highest scores (3 points), likely due to diverse patient populations and more inclusive study designs, making their results more applicable in clinical practice. Conversely, Beaudoin et al., Blaivas et al., Fletcher et al., Kang et al., Mutty et al., and Güllüpınar et al. [8-10,12-14] scored 2 points, suggesting potential limitations in generalizability due to single-center settings or restricted eligibility criteria. These findings highlight the need for larger multi-center studies to enhance the external applicability of nerve block interventions.

Bias control was strong overall, with most studies scoring 6 out of 7, except for Beaudoin et al. and Korucu et al., which scored 5 [8,10]. This suggests effective management of selection, performance, and detection biases, with only minor risks related to blinding, incomplete outcome data, or randomization procedures. Confounding control varied slightly, with scores ranging from 4 to 5. Gerlier et al., Kang et al., Mutty et al., and Güllüpınar et al. scored 5 [10-13], indicating strong statistical adjustments for baseline differences. In contrast, other studies scored 4 points, meaning they accounted for some confounders but may have had residual confounding factors affecting outcomes.

All studies scored 1 point in power analysis, confirming that sample sizes were adequately justified, reducing the risk of type II errors (false negatives). However, given that most studies had relatively small sample sizes (30-60 patients), effect size precision remains a limitation. Gerlier et al. [11] achieved the highest total score (24), followed by Kang et al. and Güllüpınar et al. [10,12] with 23 points, reflecting exceptional methodological quality. The remaining studies scored between 21 and 22, showing reliable but slightly lower quality due to minor external validity or confounding control issues.

Qualitative Synthesis of Data

The studies analyzed consistently demonstrated that ultrasound-guided nerve blocks provide superior pain relief compared to standard care. Beaudoin et al. [8] found that patients who received a femoral nerve block reported a significant reduction in pain intensity over four hours (p < 0.01), while those in the standard care group showed no significant change (p = 0.882). Furthermore, patients in the femoral nerve blocks group had a greater reduction in opioid consumption, with only five patients requiring rescue analgesia, compared to fourteen in the standard care group. Similarly, Gerlier et al. [11] reported that opioid consumption was reduced by 60% in the femoral nerve blocks group compared to the standard care group, a difference that was statistically significant (p < 0.001). This effect persisted throughout the hospital stay, with a 56% reduction in opioid use and 40% fewer opioid-related adverse events among patients who received a nerve block.

Fletcher et al. [14] further confirmed these findings by demonstrating that patients who received a 3-in-1 femoral nerve block achieved pain relief significantly faster and required less morphine per hour compared to those in the control group (p < 0.001). Mutty et al. [13] reinforced this conclusion, reporting that pain relief in the femoral nerve blocks group lasted for 12 hours on average. In contrast, the control group required more frequent morphine administration.

In the context of shoulder dislocations, studies by Blaivas et al. [9] and Raeyat Doost et al. [15] found that interscalene blocks resulted in superior pain management during reduction procedures. Blaivas et al. found that patients who received interscalene blocks required significantly fewer doses of IV fentanyl and demonstrated quicker pain relief (p < 0.05). Similarly, Raeyat Doost et al. observed that the interscalene blocks group had a 32% reduction in opioid consumption compared to the control group (p = 0.02). These studies highlight the benefits of interscalene blocks for shoulder dislocations, particularly for reducing opioid use and enhancing procedural comfort.

Kang et al. [10] compared ultrasound-guided nerve blocks to general anesthesia for femoral fractures and found that patients in the nerve block group experienced less pain during the preoperative phase and required significantly lower doses of opioids. Additionally, the nerve block group had shorter times to surgery, with a 17-minute reduction compared to the general anesthesia group (p = 0.004). Güllüpınar et al. [12] found similar outcomes in hip fractures, where pericapsular nerve group block resulted in significant reductions in both opioid use (p < 0.001) and pain scores (p < 0.01) compared to the standard care group.

Finally, Korucu et al. [16] explored the efficacy of greater occipital nerve blocks for acute migraines. They found that nerve blocks provided rapid and sustained pain relief, with 70% of patients reporting complete pain relief within one hour of treatment. This study suggests that nerve blocks may offer an effective alternative to systemic analgesics in non-traumatic pain conditions, broadening the clinical scope of ultrasound-guided nerve blocks.

The included studies reported minimal adverse effects associated with ultrasound-guided nerve blocks. The most commonly reported side effects were mild to moderate local anesthetic toxicity, which was transient and resolved spontaneously in all patients. For instance, Beaudoin et al. [8] observed that 4% of patients in the femoral nerve blocks group experienced transient numbness or tingling in the leg, which resolved within 24 hours. Similarly, Mutty et al. [13] noted that 6% of patients had transient motor weakness in the leg, but no permanent nerve injury occurred.

Other reported side effects included temporary hypotension, which was observed in up to 8% of patients, particularly those receiving femoral nerve blocks for hip fractures [8,11,13]. However, blood pressure normalization occurred without any intervention in most cases. No serious adverse events, such as infections or permanent nerve damage, were reported in any of the studies.

Discussion

The systematic review under consideration highlights the efficacy of ultrasound-guided nerve blocks in managing acute pain within emergency department settings, particularly for traumatic injuries like fractures and dislocations. This finding aligns with previous research emphasizing ultrasound-guided nerve blocks as effective alternatives to systemic analgesics. For instance, a systematic review by Gawel et al. demonstrated that when performed by adequately trained emergency physicians, ultrasound-guided nerve blocks provided effective analgesia during closed reductions of dislocated shoulders in the emergency department, with a lower risk of adverse events compared to traditional methods [17]. Similarly, a study by Beaudoin et al. found that ultrasound-guided femoral nerve blocks offered superior pain relief for hip fracture patients in the emergency department compared to parenteral opioids alone [8].

The review also underscores the role of ultrasound-guided nerve blocks in reducing opioid consumption among emergency department patients. This observation is consistent with findings from other studies. For example, a meta-analysis by Gawel et al. revealed that ultrasound-guided nerve blocks were associated with a lower risk of adverse events and complications, particularly respiratory issues, compared to procedural sedation, indicating a potential for reduced opioid use [17].

Furthermore, the review highlights the safety profile of ultrasound-guided nerve blocks, noting a low incidence of complications when performed by trained practitioners. This aligns with the findings of Amini et al., who observed that emergency physicians could safely perform ultrasound-guided interscalene nerve blocks for shoulder reductions, resulting in effective analgesia with minimal complications [18]. Similarly, the study by Gawel et al. found that ultrasound-guided nerve blocks were associated with a lower risk of adverse events and complications compared to procedural sedation [17].

The review also addresses the impact of ultrasound-guided nerve blocks on emergency department workflow, suggesting that their use may reduce the length of emergency department stays and the need for procedural sedation. This is corroborated by the study by Gawel et al. [17], which reported shorter emergency department lengths of stay for patients receiving ultrasound-guided nerve blocks compared to those undergoing procedural sedation. Additionally, Blaivas et al. [9] found that ultrasound-guided interscalene nerve blocks resulted in shorter emergency department stays and reduced provider time compared to procedural sedation for shoulder dislocations.

The review acknowledges certain limitations, including the heterogeneity of study designs, variations in ultrasound-guided nerve block techniques, and differences in outcome measures across the included studies. These factors may affect the generalizability of the findings. Moreover, the reliance on studies with small sample sizes and the potential for publication bias are notable concerns. Similar limitations have been noted in other systematic reviews on this topic. For instance, Gawel et al. [17] highlighted the need for larger, high-quality randomized controlled trials to better assess the safety and efficacy of ultrasound-guided nerve blocks in the emergency department setting.

## Conclusions

The findings from this systematic review strongly support ultrasound-guided nerve blocks as an effective and safe option for managing acute pain in the emergency department. These nerve blocks offer substantial advantages over traditional analgesic methods, including significantly reduced opioid consumption and shorter emergency department stays. By providing superior pain relief, ultrasound-guided nerve blocks help improve patient outcomes while also reducing the risk of opioid-related side effects. The safety profile of these nerve blocks is favorable, with only minor, transient side effects reported, further emphasizing their potential as a viable alternative to more invasive or systemic treatments.

However, despite the promising results, broader integration of ultrasound-guided nerve blocks into routine emergency department practice requires additional large-scale, high-quality studies. These studies should focus on establishing standardized protocols and comprehensive training programs for emergency practitioners. Such efforts will ensure the consistent and optimal use of nerve blocks, ultimately allowing them to become a routine part of acute pain management strategies in emergency care. Through this, patients can benefit from enhanced pain relief while minimizing the need for opioids and the associated risks.
